# Analysis of the research subjects and hot topics of occupational diseases through the Web of Science from 1975 to 2021

**DOI:** 10.3389/fpubh.2022.1009203

**Published:** 2022-09-08

**Authors:** Hamid Reza Saberi, Hadiseh Rabiei, Asma Zare, Milad Derakhshan Jazari, Mahdi Malakoutikhah

**Affiliations:** ^1^Social Determinants of Health (SDH) Research Center, and Department of Occupational Health Engineering, Kashan University of Medical Sciences, Kashan, Iran; ^2^Occupational Health Engineering, Department of Occupational Health Engineering, School of Health, Shahid Beheshti University of Medical Sciences, Tehran, Iran; ^3^Department of Occupational Health, Sirjan School of Medical Sciences, Sirjan, Iran; ^4^Occupational Health and Safety Engineering, Department of Occupational Health Engineering, School of Health, Baqiyatallah University of Medical Sciences, Tehran, Iran; ^5^Occupational Health and Safety Engineering, Department of Occupational Health Engineering, School of Health, Kashan University of Medical Sciences, Kashan, Iran

**Keywords:** occupational disease, scientometrics, respiratory disease, epidemiology, occupational health

## Abstract

A variety of studies have been conducted in Occupational diseases (ODs) and this makes it difficult for researchers to identify new areas of study. Therefore, the present study was conducted by examining Web of Science data to identify hot topics and research topics on ODs. This is a scientometric study performed using CiteSpace and Gephi software for statistical analysis. The published article in Web of Sciences was searched using the keywords “Occupational disease^*^” OR “Occupational illness^*^” OR “Industrial disease^*^” OR “Industrial illness^*^”. Finally, the countries and institutions and their cooperation, the most important and main topics discussed, and the path of future progress in ODs was analyzed. Preliminary results of the study show that out of 5,947 articles. The results of important journals showed that the American Journal of Industrial Medicine with 233 articles (6.02%), Oxford Occupational Medicine, and International Archives of Occupational and Environmental Health with 86 (2.22%), and 83 (2.15%), respectively. The two producing countries are the United States and Germany, which published 628 and 419 articles, respectively. The results of hot topics showed occupational exposures, epidemiology, mental health, and respiratory diseases were the most important keywords used in these 45 years. It can be concluded that Germany, with its current development trend in the coming years, will surpass the United States based on the number of articles and gain the first rank. Also, future studies can be conducted on respiratory diseases as the most important ODs and health care work as the most important job during the past years.

## Introduction

Occupational diseases (ODs) can be defined as an illness caused or aggravated by work. The International Labor Organization (ILO) defines ODs as diseases caused by exposure to risk factors of work activities (1). The World Health Organization (WHO) states that an ODs is not defined solely by the disease itself; it is a combination of an illness and its exposure, as well as the relationship between these two norms (2). In another definition, “ODs” are classified into two categories; one is traditional ODs that can be caused only by occupational reasons caused by exposure to chemicals, physical and biological agents. Another case is work-related illnesses that can be exacerbated, stimulated, or affected by work conditions. These are musculoskeletal diseases (MSDs) or cardiovascular diseases (CVDs) that are caused by several factors, including the type of occupation (3).

Estimates for the global incidence of ODs vary considerably. Historically, Alaguney has predicted 4 to 12 ODs per 1,000 employees (4), and Leigh et al. have predicted 4 to 10 million ODs per year (5). The ILO estimates that there are 160 million non-fatal diseases and 2 million deaths a year (6), which account for at least 4% of global gross domestic products or about $2.8 trillion in direct and indirect costs (7). ODs can also reduce the standby time of manpower. They can lead to the rapid elimination of labor from the labor market, causing high-consumption members of society (8). It is predicted that by 2050, about a third of the workforce will have been over 50 years old (9), and this is likely to lead to a sharp decline in labor productivity. In addition, if no one pays attention to the current occupational health of the workforce, it can impose more costs on the governments to health care systems in the future.

These statistics show ODs in the world. Therefore, concerning ODs, studies have been conducted in this field, and as far as the types of diseases have been studied, the study of the risk and burden of the disease helps the authorities to identify and control this disease as much as possible. Because the prevalence of ODs is changing due to the changes in industry and working conditions, the list of ODs has been reviewed both in various studies and in the ILO. This revision has been influenced by the modernization of industry and other international organizations as well as the European Union; it has also been influenced by the development and revision of the National Job List. Therefore, due to the very high number of studies in the field of ODs, the need for these studies has been observed and its important cases are reported to researchers who are studying in this field. In this regard, some studies have reviewed some specific occupational diseases, for example, studies such as “Historical review of the list of ODs recommended by the International Labor Organization” by Kim et al. (6), review studies in the field of specific ODs such as isocyanate (10), a review study of ODs in small and medium industries in Malaysia (11), and the most important ODs in the construction industry (12) have been conducted. However, it is not possible to get study ideas from these studies, and it is not possible to examine and identify hot topics, frequently used keywords, institutions and countries that have conducted the most studies in this field. Therefore, it is necessary to clarify the path of future studies with a scientometric study and provide researchers with results that can better shape their next studies.

Accordingly, with an overview of different studies, it shows that different studies have been conducted over the years and the number and variety of studies in this field is very high and can confuse the researchers; therefore, identifying the most important areas of study for ODs seems to be difficult. Therefore, the present study was conducted by examining Web of Science data to identify hot topics and research topics on ODs in a scientometric study. The present study can predict future development directions by stating the countries, institutions, and publications about ODs and analyzing the frequency of keywords.

## Materials and methods

This is a scientometric study performed using CiteSpace and Gephi software for statistical analysis of citations from scientific sources (13). The first step in a scientometric study is to identify and extract studies in the field under study. Therefore, the search was performed using the keywords and the OR command in the form of “Occupational disease^*^” OR “Occupational illness^*^” OR “Industrial disease^*^” OR “Industrial illness^*^”. All published studies related to the selected keywords on the Web of Science from 1975 to 31/12/2021 were included in the study. The reason for choosing the start date of the studies was the time limit of the Web of Sciences for searching studies.

Finally, in this study, we aimed to create a good plan for future studies and also introduce new study topics for researchers and help them find collaborating partners. Therefore, the following questions were posed:

1) Which countries and institutions study ODs? And who are their colleagues?
2) What are the most important and main topics discussed? What are the most important current issues?
3) What is the path of future progress in ODs?

The database created also included:

- Science Citation Index Expanded (SCI-EXPANDED)
- Social Sciences Citation Index (SSCI)
- Conference Proceedings Citation Index–Science (CPCI-S)
- Conference Proceedings Citation Index–Social Science and Humanities (CPCI-SSH)
- Current Chemical Reactions (CCR-EXPANDED)
- Index Chemicus (IC).

The study was divided into the following five stages:

Step 1: Data were collected through Web of Science according to the search query. The research result was then extracted and saved in the text format recognizable by CiteSpace.
Step 2: The data were cleared. First, synonym keywords were merged. Second, meaningless words like related words and meaningless nouns were removed.
Step 3: The research results were entered into CiteSpace, Gephi, and VOS viewer software. CiteSpace software was used for the initial review of articles; Gephi was used to map the relationships and collaborations, and VOS viewer software was used to analyze the keywords.
Step 4: The research content required for analysis and duration in the software were selected.
Step 5: Graphs and visualization of the results were performed.

After determining the search formula and databases, 5,947 articles related to the purpose of the study were obtained and the information about author, title, publication sources, references, keywords, and abstract was extracted.

## Results and discussion

Preliminary results of the study show that out of 5,947 all type of articles, 44,000 were orginal papers, 557 were Review articles, 568 were Proceeding Paper, and 112 were Letter reports. Of 5,947 articles, 4,450 were in English and the rest were in other languages (624 German studies, 305 French studies, 162 Spanish studies, 117 Portuguese studies, 52 Italian studies, 57 Russian and Polish studies, 38 Turkish studies, 24 Ukrainian studies, 10 Chinese studies, 9 Korean studies, 8 Czech studies, 4 Hungarian studies, 2 Hungerian studies, Dutch, Persian, Slovak, Slovenian and Greek each had 2 studies and Arabic, Galician, Indonesian, Latvian, Swedish each had 2 studies.). Also, the first studies were published in 1975 with 19 studies and since then it has had a growing trend.

**Figure 1:**
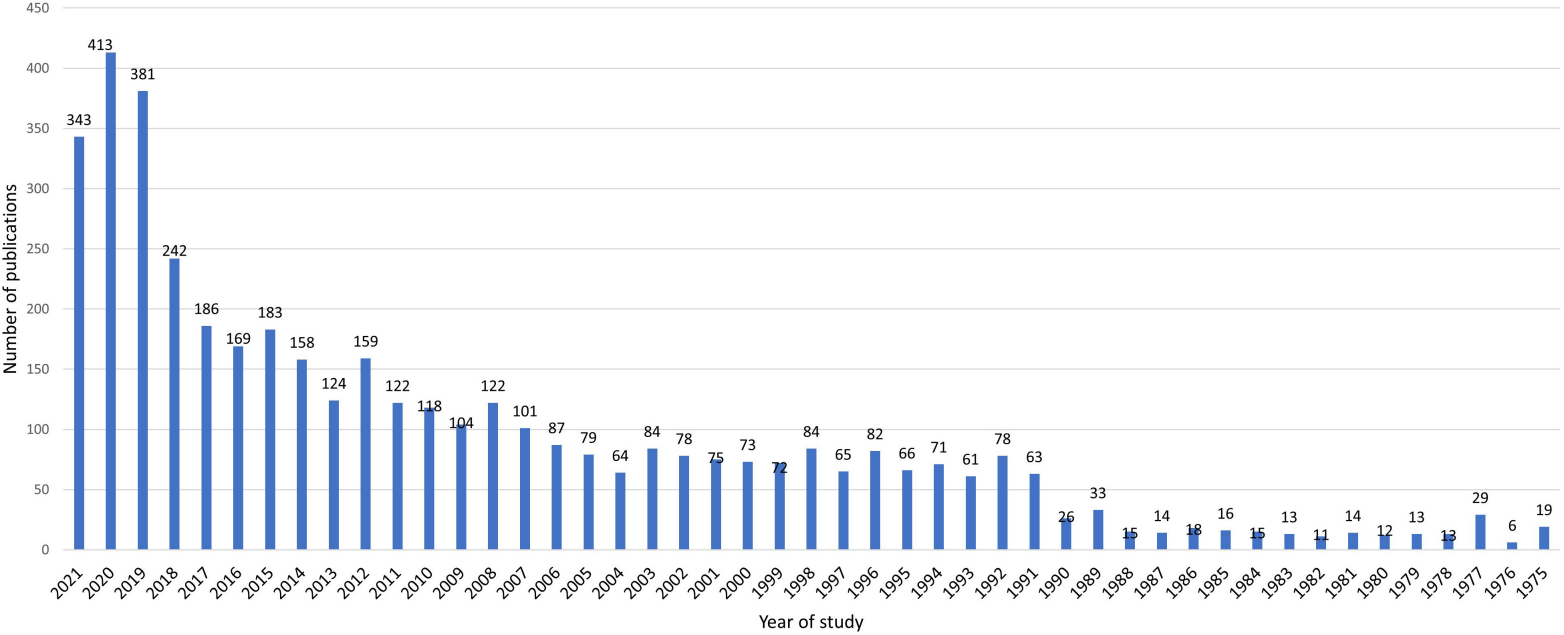
The process of publishing articles on occupational diseases over the past 46 years.

Over time, the number of studies until 1991 was under 50 studies per year, and since 1991, with 63 studies, an increasing trend of studies can be seen; also, since 2007, more than 100 studies have been published each year. The highest number of studies was done in 2020 (Figure 1).

The process of publishing studies in terms of citations based on different years can show the importance of the subject of ODs during different years and helps to better examine the keywords and identify important topics of recent years. The results of this section based on the analysis of articles over three 15-year periods from 1975 to 1990, 1991 to 2006, and 2007 to 2021 in Cite Space software showed that in the first 15 years (1975–1990) 279 citations, in the second 15 years 25,767, and during the third 15 years 15,303 references to all articles have been made.

## The most cited studies in the field of ODs

The results of the most cited studies show the importance of an issue in the field of ODs, as shown in Table 1. The article by Grossi et al. (14) had the most citations among different studies (750 citations). This article was published in 1994 in the Journal of Periodontology. The second study was Tong et al., that published in Bulletin of the world health organization and it is about occupational and environmental lead exposure. This paper showed that both occupational and environmental exposures to lead remain a serious problem in many developing and industrializing countries. Acute lead poisoning has become rare in such countries, but chronic exposure to low levels of the metal is still a public health issue. Also, the results of the average citation per year of studies show that the study of Eisner et al. (16), although published in 2010, has 543 citations and is the best study in this regard. This study is about identifying new risk factors and the global burden of chronic obstructive pulmonary disease; since respiratory diseases are the most important ODs (24), this study can help prevent this type of disease by identifying new risk factors. Therefore, it can be concluded that the importance of respiratory diseases, especially chronic obstructive pulmonary disease, is very high in ODs studies.

**Table 1:** The most cited studies in the field of occupational diseases.

**Authors**	**Journals**	**No. Publicati**	**Citations**	ACPY*	**References**
Grossi et al.	Journal of Periodontology	1994	750	26.79	(14)
Tong et al.	Bulletin of the World Health Organization	2000	579	26.32	(15)
Eisner et al.	American Journal of Respiratory and Critical Care Medicine	2010	543	45.25	(16)
Warrell	The Lancet	2010	404	33.67	(17)
Spelten et al.	Psycho-Oncology: Journal of the Psychological, Social Behavioral Dimensions of Cancer	2002	394	19.7	(18)
Pruss-Ustun et al.	American Journal of Industrial Medicine	2005	375	22.06	(19)
Gottschalk et al.	Future Microbiology	2010	294	24.5	(20)
Theriault et al.	American Journal of Epidemiology	1994	273	9.75	(21)
Cohen et al.	Journal of the American Dental Association	1980	271	6.45	(22)
Hunt	Journal of Allergy and Clinical Immunology	2002	246	12.3	(23)

^*^ACPY, Average Citations per Year.

## The best journals and the top area in the field of ODs

The results of journals that have published articles in this field from the Web of Science show that a total of 500 journals have published articles related to ODs, of which the top 10 journals published a total of 747 articles. The results of the study show that the American Journal of Industrial Medicine with 233 articles (6.02%) has published the most articles in this field. Oxford Occupational Medicine, International Archives of Occupational and Environmental Health, and Journal of Occupational and Environmental Medicine have the highest number of articles with 86 (2.22%), 83 (2.15%), and 62 studies (1.60%), respectively (Table 2). Also, based on the number of citations, it can be seen that the American Journal of Industrial Medicine had the most citations and citations ratio.

**Table 2:** Top 10 journals with the highest number of published studies on occupational diseases.

**No**.	**Journal name**	**Impact factor** **(in 2021)**	**Number of published studies**	**Number of citations**	**The ratio of citations to published studies**
1	American Journal of Industrial Medicine	2.214	233	5,782	24.82
2	Occupational Medicine Oxford	1.611	86	1,180	13.72
3	International Archives of Occupational and Environmental Health	3.015	83	1,262	15.20
4	Journal of Occupational and Environmental Medicine	2.162	62	1,485	23.95
5	Archives Des Maladies Professionnelles Et De L Environnement	0.205	53	50	0.94
6	Medicina Del Lavoro	1.275	48	160	3.33
7	Occupational And Environmental Medicine	4.402	48	1,133	23.60
8	Revue Des Maladies Respiratoires	0.622	47	200	4.26
9	Medycyna Pracy	0.760	44	165	3.75
10	International Journal of Environmental Research and Public Health	3.309	44	298	6.93

**Figure 2:**
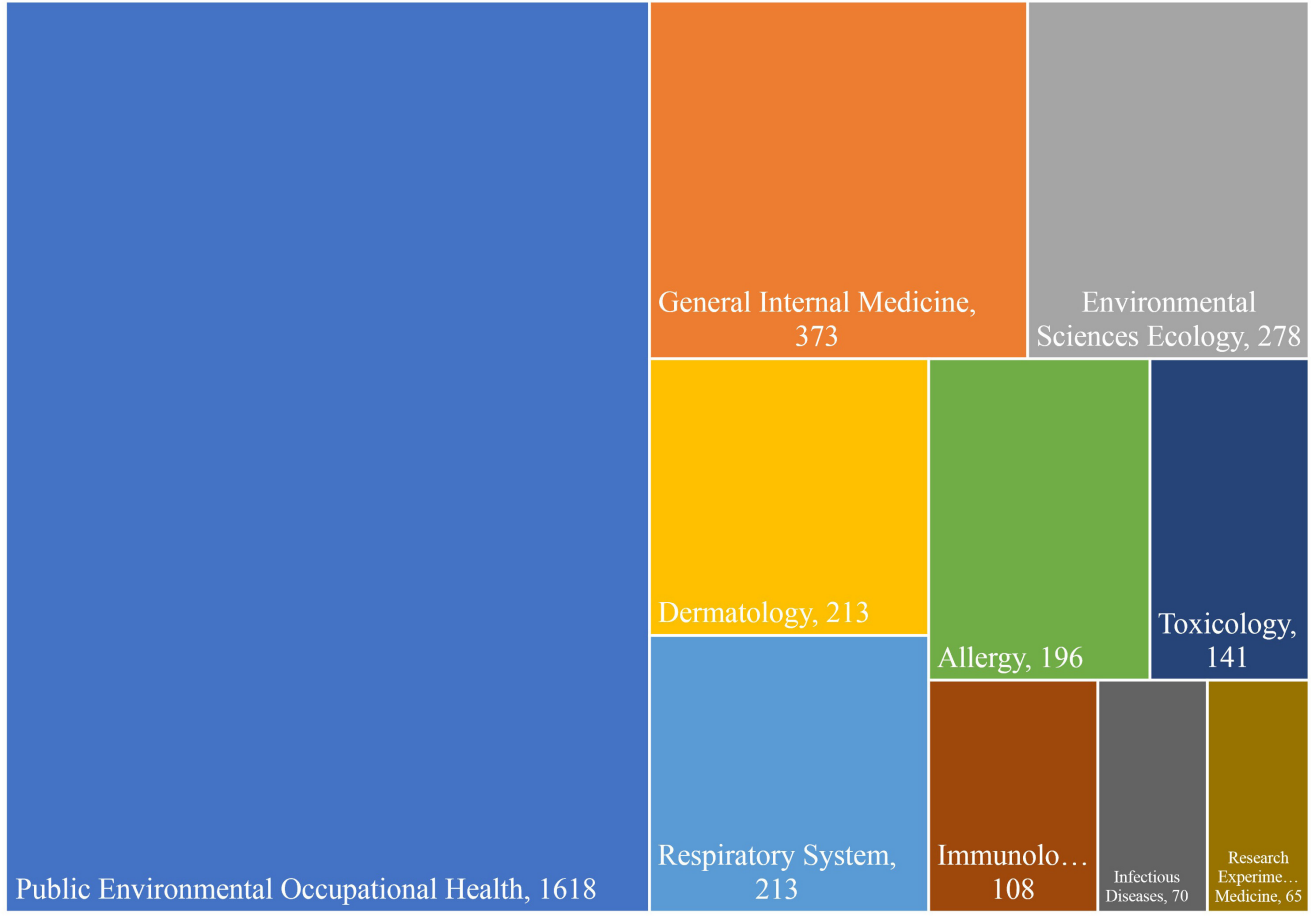
Top 10 specialized areas of publishing articles on occupational diseases over the past 46 years.

A review of the top 10 specialized areas on which studies have been conducted shows that the field of Public Environmental Occupational Health with 1,618 studies is the most important area, and followed by General Internal Medicine with 373 studies and Environmental Sciences Ecology with 278 studies (Figure 2).

According to Figure 2, the most important area was Public Environmental Occupational Health. This area generally deals with occupational health and environmental diseases, of which respiratory diseases are the most studied, followed by various cancers (Table 3). As can be seen, this field has been studied separately since 1991 and has dealt with ODs in general. Lung diseases are known as the most common ODs so that about 40% of ODs are related to lung diseases (25). Although thousands of chemicals are used worldwide without any proper testing, a small fraction have been evaluated, at least in part, for carcinogenicity. However, despite a fully established classification by the International Agency for Research on Cancer (IARC), monitoring of occupational exposure to known carcinogens is often lacking nationally. This knowledge gap has been repeatedly cited as a major constraint on occupational cancer prevention and the assessment of occupational cancer burden, which may be addressed (26). In addition to the diseases mentioned in the last 2 years with the pandemic of COVID disease 19, studies of this disease have also been reviewed from an occupational point of view, and in the field of Public Environmental Occupational Health, 15 studies have been reviewed.

**Table 3:** Diseases studied in domain Public Environmental Occupational Health by study period.

**Diseases studied**	**No. studies**	**Years**		
		**1975–1990**	**1991–2006**	**2007–2021**
Respiratory diseases	282	0	275	7
Cancer	181	0	175	8
Musculoskeletal disorders	126	0	109	17
Occupational diseases	65	0	57	8
Skin diseases	50	0	40	10
Cardiovascular diseases	36	0	24	12

## Top 10 producing countries, cited countries and analysis of cooperation in the field of ODs

**Figure 3:**
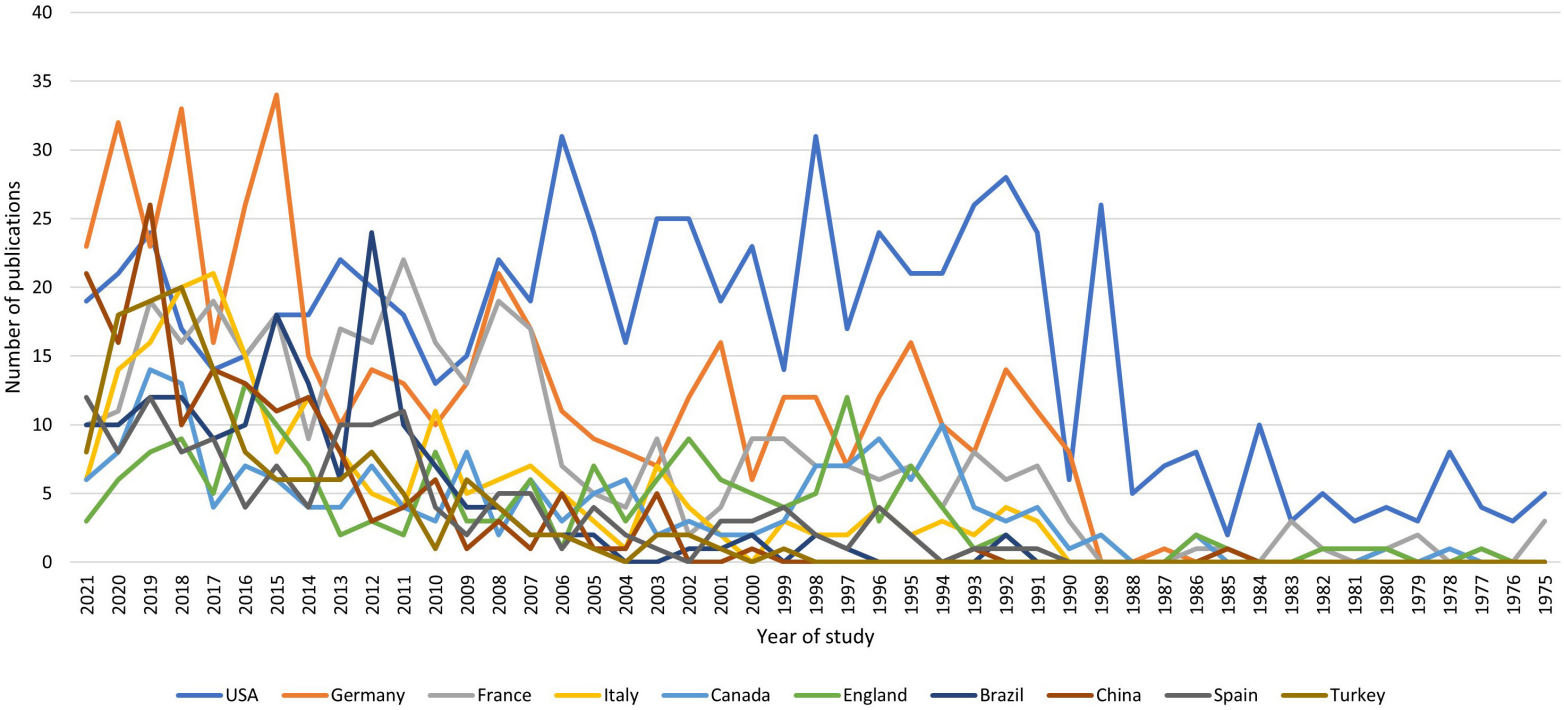
Top 10 countries which published articles on occupational diseases over the past 46 years.

Using the Web of Science results, we extracted the top 10 countries (Figure 3). The two producing countries are the United States and Germany, which published 628 and 419 articles, respectively. In addition, a review of the first years in which articles on ODs were published shows that the two countries initiating these studies were the United States and France, respectively, which published the article in 1975. Germany, which ranks second in terms of the number of articles, published its first study in 1987, and over time, since 1990, has increased its number of studies to second place. The highest number of studies in this country was in 2015 with 34 studies. This shows that the growth rate of German articles is very high.

Among the top 10 countries are eight developed countries that have published 2,440 articles. However, the two developing countries have published 304 articles. This shows that there is still a large gap between developed and developing countries in ODs research.

Examining the growing curve of Germany and the United States can predict the trend of publishing articles in the next 2 years. After obtaining the annual emission values of the two countries, MINITAB was used to analyze the cumulative growth curve regression. The best fit adj-R square curve for the United States is 0.341, while for Germany it is 0.721; this indicates that the trend in German article production in the coming years will be much higher than in the United States. The closer the adj-R squares to 1, the better the curve.

**Figure 4:**
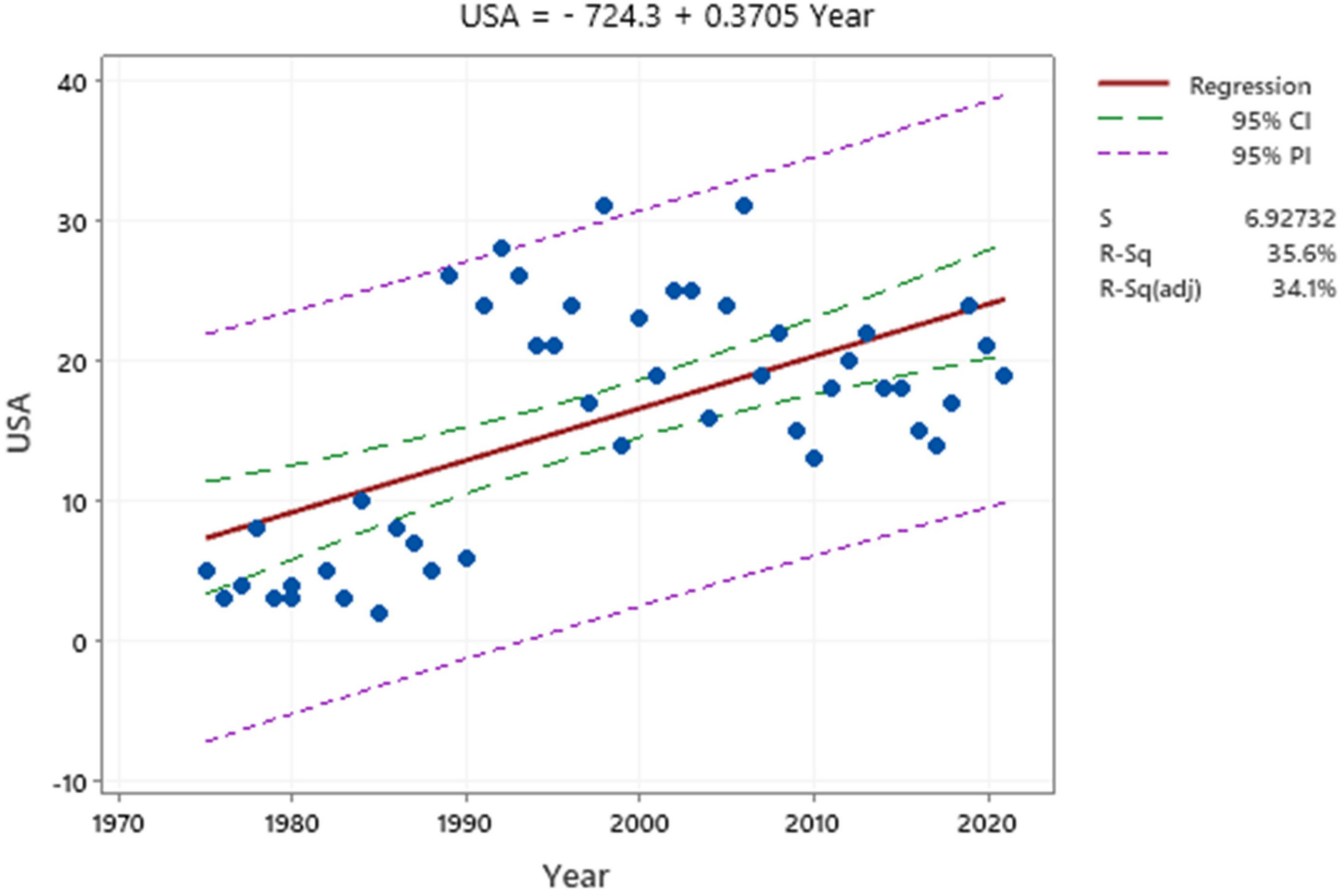
Article production curve in the United States.

**Figure 5:**
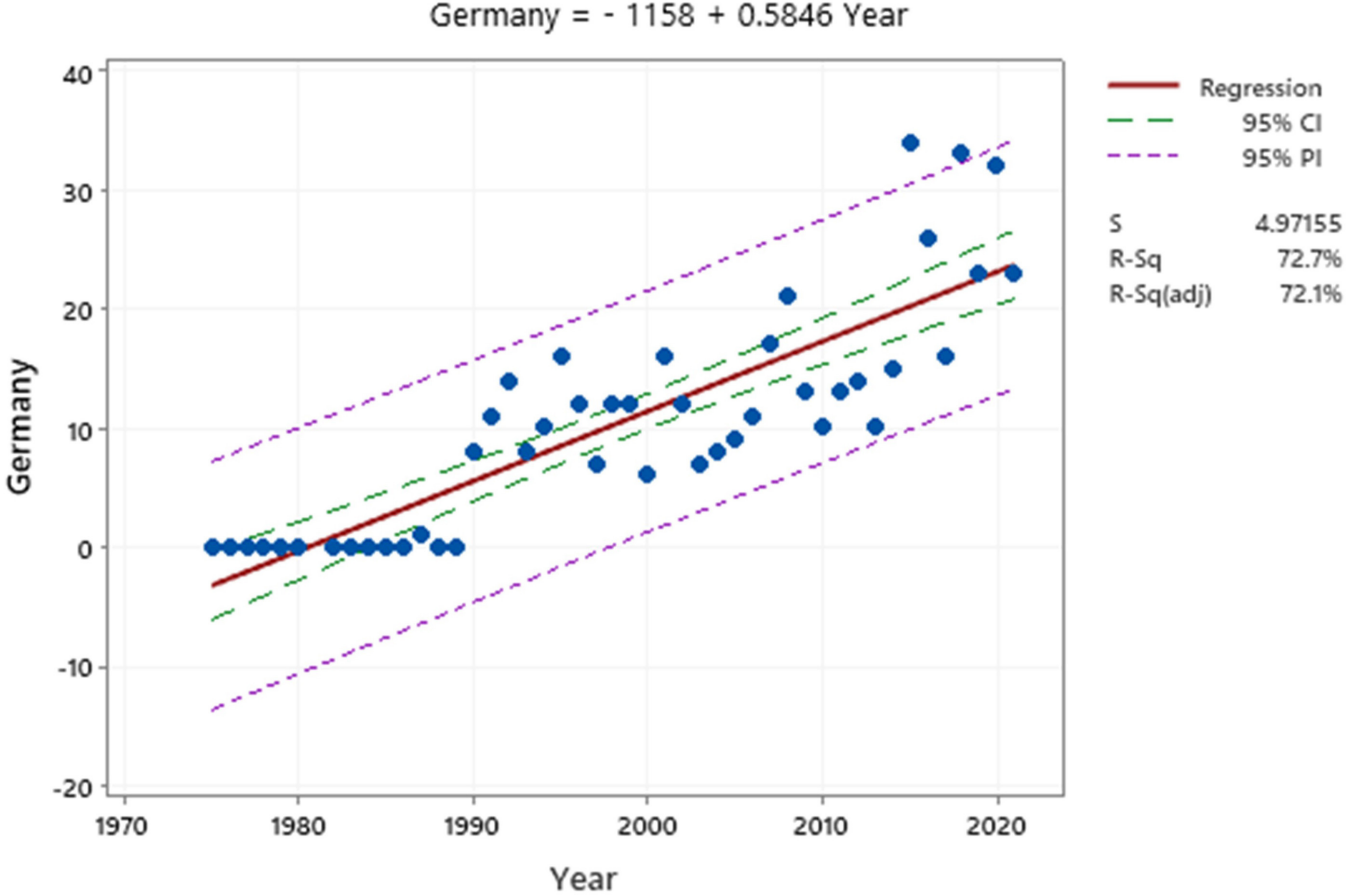
Article production curve in Germany.

The best-suited curves for the United States and Germany are shown in Figures 4, 5. A comparison of Figures 4, 5 shows that the growth rate in Germany is higher than in the United States. This shows that Germany has published more studies on ODs. The number of emissions in the United States was higher than in Germany before 2008, but Germany is developing at a faster pace, while the slope of the chart for the United States is almost constant. The upward trend in the United States began in 1975 and peaked in 1998 and 2006. However, the upward trend in Germany started in 1990 with 8 studies and reached its peak in 2015 with 34 studies.

The results of the Web of Science site showed that 100 countries had studied ODs. The analysis of cooperation relations between the countries shows that Spain (144%), the United Kingdom (123%), and Italy (97%) have higher rates of international cooperation in the top 10 producing countries. This shows that the three countries pay more attention to communication with other international countries (Table 4). Spain published 141 studies and 203 collaborations, showing that all of its studies are produced in collaboration with other countries. In contrast, Brazil and Turkey rank 9th and 10th, respectively, with 17 and 13% of international cooperation, respectively, preferring foreign studies as domestic teamwork. Therefore, it can be concluded that cooperation relations in developed countries are more than in developing countries.

**Table 4:** Top 10 countries in producing studies on occupational diseases.

**Country**	**No. publications**	**No. cooperation**	**International cooperation rate**	**Centrality**
USA	746	217	0.29	0.44
Germany	480	196	0.41	0.06
France	354	222	0.63	0.06
Italy	206	200	0.97	0.05
Canada	179	128	0.72	0.08
England	170	209	1.23	0.13
Brazil	164	28	0.17	0.01
China	164	39	0.24	0.01
Spain	141	203	1.44	0.05
Turkey	140	18	0.13	0.01

Table 4 also shows the centrality index for all countries. This index indicates the importance of the position of a node in a determination network (27). It measures the value of each of the central nodes in the path connecting to other network nodes based on the shortest path (28); the value of centrality has no role if the node is < 0.1. If it is greater than or equal to 0.1, it has a strategic position, in which case it can be a candidate for a turning point, and if it is > 1, it is a turning point (critical) and will have a unique position. As the Table shows, only two countries, the United States and the United Kingdom have more centrality (0.1), and the United States, with a centrality of 0.44, has the largest role in occupational disease studies and can be considered as a country with a strategic position in the field of ODs.

**Figure 6:**
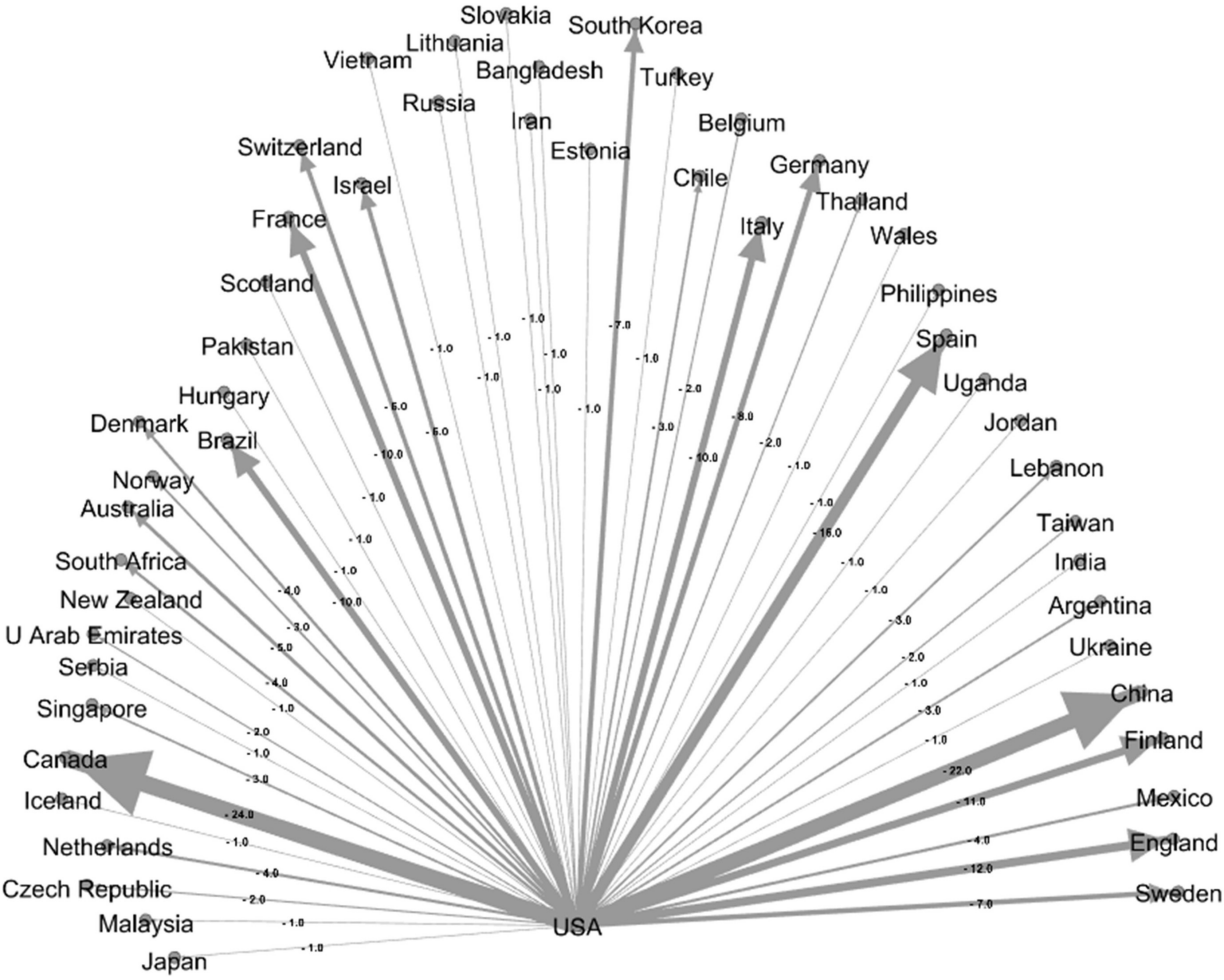
US cooperation network with other countries.

**Figure 7:**
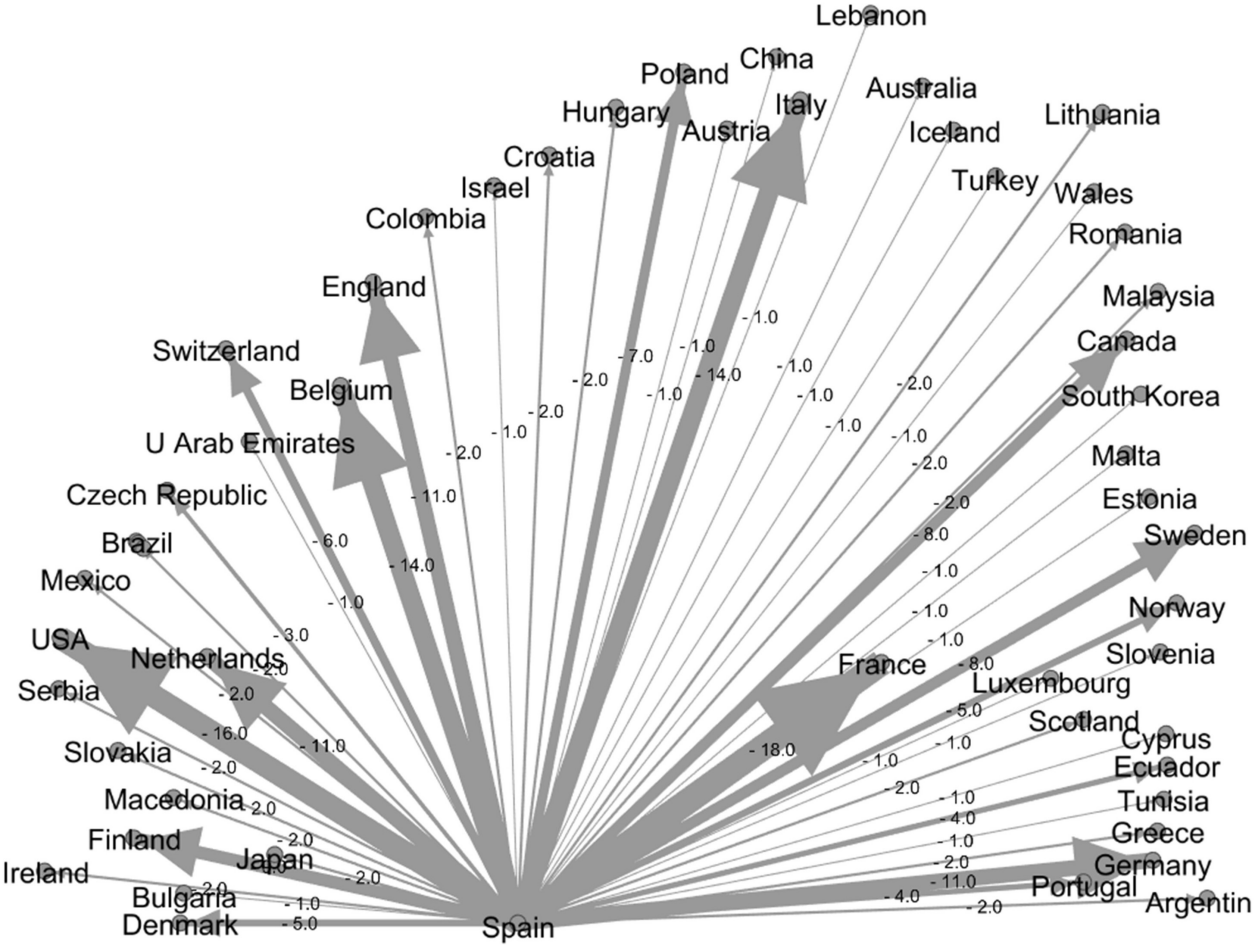
Spain cooperation network with other countries.

Figures 6, 7 show the US cooperation network as the country with the most productivity and Spain as the country with the most cooperation, respectively. As can be seen, these two countries have cooperated with 50 countries each. Gephi software was used for this purpose. According to this software, the thicker the connection line, the more cooperation the two countries have with each other. As Figure 6 shows, the United States has had the most cooperation with Canada with 24 articles, followed by China with 22 articles. Spain also has an extensive cooperation network, with 18 studies having the most cooperation with France.

One way to identify a country's international impact on its science production is the number of times it is cited. The top 10 countries in terms of the number of citations are shown in Table 5. As can be seen, the United States has not only the highest number of citations per article but also the highest number of citations, with an average citation rate of 22 per article from 1975 to 2021 and a total of 16,504 citations with 746 citations. Canada is the fifth most published country and the third most cited country. This means that Canada also has a broad scientific impact on the study of ODs. Comparing the top 10 countries in publication with those in the citation, we can be concluded that 7 of the top 10 countries in the publication are among the top 10 countries in terms of citation, and Flanders, the Netherlands, and Sweden, despite being among the top 10 countries, are not the top producers of articles; they are among the top 10 countries cited. Sweden is the top country in terms of average citations with 68 articles published and 1,877 citations and an average of 28 citations per article. However, Germany and France are lower than other countries by an average of 8 citations per article.

**Table 5:** Top 10 countries on occupational diseases based on citations.

**Top 10 productive countries**				**Top 10 highly cited countries**			
**Country**	**No. publications**	**Cited frequency**	**Average citations**	**Country**	**No. publications**	**Cited frequency**	**Average citations**
USA	746	16,504	22	USA	746	16,504	22
Germany	480	3,961	8	Germany	480	3,961	8
France	354	2,698	8	Canada	179	3,225	18
Italy	206	2,259	11	Finland	112	2,979	27
Canada	179	3,225	18	France	354	2,698	8
England	170	2,632	15	England	170	2,632	15
Brazil	164	1,132	7	Italy	206	2,259	11
China	164	1,758	11	Netherlands	92	1,959	21
Spain	141	1,706	12	Sweden	68	1,877	28
Turkey	140	650	5	China	164	1,758	11

## Top 10 cited institutions and collaboration analysis

Institutions that have studied ODs can also be important. The results showed that 500 institutes had studied in this field. Also, cooperation in the production of studies is not limited to countries and is also formed among institutions. The authors' address published in a study is considered as an institution and their collaboration. The top 10 institutions in the field of ODs along with their cooperation with all institutions are shown in Table 6. The results show that 3 institutes are from Germany and confirm the upward trend of studies mentioned in the previous section. Only the two institutions in the United States with the highest number of articles are in the top 10 ranks. Also, three countries, Flanders, Poland, and the Netherlands, which were not among the top 10 countries in terms of the number of articles, with the participation and cooperation of their institutions, are among the top 10 institutions in terms of the number of articles, which shows the role of international cooperation in article production. The results of the cooperation showed that the Osnabrück University from Germany and the Finnish Institute of Occupational Health from Flanders had the most cooperation. The collaboration rate shown in Table 4 is the ratio of the number of collaborations to the number of articles published, which shows that the institution has had more collaborations. Osnabrück University from Germany had the most contributions with 35 papers and a collaboration rate of 2.83. Therefore, transnational studies can be done with this institute.

**Table 6:** Top 10 institutions in producing studies on occupational diseases.

**Institutions**	**Country**	**No. publications**	**No. cooperation**	**Cooperation rate**
National Institute for Occupational Safety and Health (NIOSH)	USA	87	98	1.13
Finnish Institute of Occupational Health	Finland	65	99	1.52
University of Toronto	Canada	47	89	1.89
Nofer Institute of Occupational Medicine (NIOM)	Poland	43	98	2.28
University of Amsterdam	Netherlands	38	79	2.08
University of Erlangen-Nuremberg	Germany	38	26	0.68
Ruhr-University Bochum	Germany	35	86	2.46
Osnabrück University	Germany	35	99	2.83
University of Washington	USA	33	50	1.52
Inserm Research institute	France	32	15	0.47

To find the most effective institutions in the field of ODs, the results of cited institutions among the top 10 institutions were analyzed (Table 7). As the results show, there are 5 institutions from the United States, which were only 2 institutions from the top 10 ones in this country, and this shows the importance of cooperation between institutions. On the other hand, in Germany, where three institutions were among the top 10 institutions in terms of the number of articles, there is no institution among the top 10 institutions cited. One institution from Belgium is also one of the 10 cited institutions although Belgium was not among the top 10 countries in the number of articles cited. The top institution in terms of average citations is the University of North Carolina at Chapel Hill, which has 1,562 citations with 25 studies. After that, the University of Massachusetts Institute with 19 studies and 907 citations has had the greatest impact on the study of ODs. This shows that although these two institutes are not among the top 10 institutes in terms of the number of studies, the type of their studies and objectives of their studies are fundamental and form the basis of subsequent studies.

**Table 7:** Top 10 institutions on occupational diseases based on citations.

**Institutions**	**Country**	**No. publications**	**Cited frequency**	**Average citations**
National Institute for Occupational Safety and Health (NIOSH)	USA	87	2,710	31.15
Finnish Institute of Occupational Health	Finland	65	1,901	29.25
University of North Carolina at Chapel Hill	USA	25	1,562	62.48
University of Toronto	Canada	47	1,150	24.47
Catholic University of Leuven	Belgium	28	940	33.57
Inserm Research institute	France	32	934	29.19
Montreal Sacred Heart Hospital	Canada	22	927	42.14
University of Massachusetts	USA	19	907	47.74
Michigan State University	USA	19	906	47.68
Harvard University	USA	25	855	34.20

Also among the high citation studies, in addition to the studies listed in Table 1, three studies entitled Negative Impacts of Shiftwork and Long Work Hours with 218 citations from NIOSH (29), Cancer Risk Associated with Occupational Exposure to Magnetic-Field among Electric Utility Workers in Ontario, Quebec, Canada, and France, 1970–1989 with 273 citations from the University of Toronto (21), and the Work-Related Cumulative Trauma Disorders Study of the Upper Extremity with 229 citations from the University of Washington (30) also had the most citations.

### Important topics in the field of ODs

Important keywords and topics usually reflect the current research trends and provide authors with suggestions for future studies. By analyzing the keywords in Cite Space software, we can extract the top 10 keywords by classifying them into three periods: 1975 to 1990, 1991 to 2006, and 2007 to 2021. According to the results in Table 8, the keywords of the studies can be classified into three categories of keywords related to the types of ODs, the studied jobs, and the epidemiological studies. This Table shows that in the first period, the words Occupational exposures, Epidemiology, and Occupational diseases ranked first to third, and during the second period, Skin diseases replaced Occupational diseases in the third place. Also, the term Mental health, which was not used at all from 1975 to 1990, has become the second most frequently used term in the period 2007 to 2021; this shows the importance of this issue in the study of occupational diseases. Respiratory Diseases, which are the most important occupational diseases, have been ranked fourth among the keywords in the three study periods and have always been of great importance, showing more jobs than before. Studies have also shown that lung disease has declined in recent years, and ODs including CVDs and MSDs have increased since 1996 after diagnostic criteria were determined. From 1996 to 2009, low back pain accounted for 41.2% of the work-related illnesses, followed by CVDs at 30.3% and MSDs (excluding low back pain) at 25.2% (3).

**Table 8:** Repetition of keywords over the last 15 years periods (from 1975 to 2021).

**1975–1990**		**1991–2006**		**2007–2021**	
**Keywords**	**Frequency**	**Keywords**	**Frequency**	**Keywords**	**Frequency**
Occupational exposures	1,566	Occupational exposures	209	Occupational exposures	47
Epidemiology	902	Epidemiology	147	Mental health	46
Occupational diseases	596	Skin diseases	85	Jobs	44
Respiratory Diseases	451	Respiratory Diseases	72	Respiratory Diseases	40
Cancer	230	Musculoskeletal disorders	70	Epidemiology	39
Occupational accidents	166	Jobs	57	Musculoskeletal disorders	35
Allergy	147	Mental health	37	Cancer	24
Musculoskeletal disorders	131	Occupational diseases	32	Cardiovascular diseases	22
Jobs	106	Cancer	30	Noise and vibration diseases	16
Skin diseases	76	Occupational accidents	17	Occupational accidents	9
Cardiovascular diseases	14	Allergy	5	Skin diseases	6
Noise and vibration diseases	4	Noise and vibration diseases	1	Occupational diseases	2
Mental health	0	Cardiovascular diseases	0	Allergy	2

**Figure 8:**
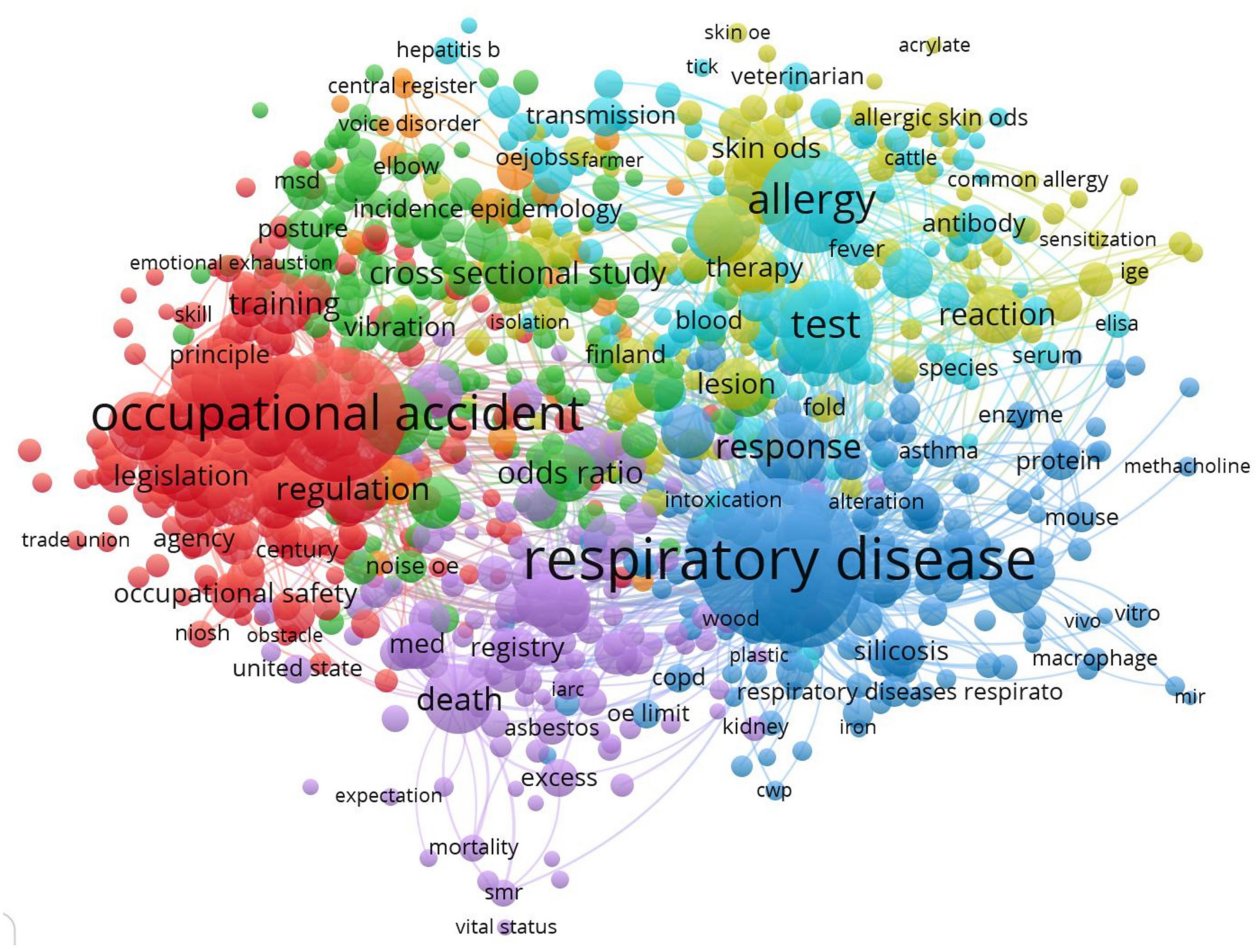
Keyword communication network.

Figure 8 shows the relationship between the keywords, which is plotted using VOS viewer software. This connection means that the term A is related to the term B; in addition, the term B is related to the term C. It can be concluded that there is a relationship between term A and term C, and this further promotes the scientific findings. Therefore, the analysis of the simultaneous relationship between keywords can raise a new ground in the study of ODs. Figure 8 shows that respiratory diseases, occupational accidents, and allergies are widely used in ODs. Based on the definition of ILO for occupational accidents “an occupational accident is an unexpected and unplanned occurrence, including acts of violence, arising out of or in connection with work, which results in one or more workers incurring a personal injury, disease or death.” (31), Since, it can lead to disease, this keyword has been used in studies. Of course, there is a difference between occupational disease and occupational accidents.

To better understand the keywords, we examined Occupational exposures, Epidemiology, Respiratory Diseases, Mental health, and Jobs in detail due to their frequency during different periods. The results of the subcategories of the keyword Occupational exposure showed that this term could be classified into four categories of Exposure, Workplace exposure, Risk factors, and Occupational health. These four words were repeated 420, 478, 417, and 251 times in the first period, respectively; 72, 87, 52, and zero times in the second period; and 7, 7, 13, and 20 times in the third period, respectively. This indicates that workplace exposure is of great importance during the study period, after which the importance of exposure time has been removed and most of the risk factors for ODs have been studied. Therefore, in the field of occupational health in the coming years, researchers can further investigate the risk factors. The keyword Mental Health has been added to those of occupational disease since 2004 and includes items such as stress, burnout, depression, and anxiety, which have increased significantly during the years 2021–2007.

Epidemiological studies in this field can also be divided into several categories (Table 9). The results showed that the study of Prevalence, Mortality, Surveillance, Epidemiology, and Follow up were the most frequent words in the first 15 years, but over time the repetition of these words has decreased and in recent years only the words Mortality and Burden of diseases are important.

**Table 9:** Keywords related to epidemiology.

**Keywords**	**1975–1990**	**1991–2006**	**2007–2021**
Prevalence	229	0	6
Mortality	143	0	13
Surveillance	86	58	0
Epidemiology	173	45	0
Follow up	103	0	2
Intervention	11	0	0
Burden of diseases	27	0	11
Others	130	44	7

As the results of the top areas (Figure 2) and keywords (Table 8) showed, and also based on other studies, respiratory diseases are the most important ODs (32); therefore, respiratory diseases were examined in detail (Table 10). As Table 10 shows, Chronic obstructive pulmonary disease (COPD) has had the highest recurrence among lung diseases over the past 4 decades because it covers a wide range of lung diseases. This category includes words such as lung disease, atherosclerosis, respiratory health, airway inflammation, airways obstruction, and so on. After this disease, occupational asthma was very important from 1975 to 1990, which has decreased in recent years. The results also showed that asbestosis was prevalent from 1975 to 1990, but, with the application of the asbestos removal law, this keyword has not been seen in ODs in recent years. Pneumoconiosis is one of the most common respiratory diseases today, which shows the importance of this disease in recent years. These results are in line with those of previous studies which show that before 1991 most cases of ODs were pneumoconiosis, and since 1991 the proportion of pneumoconiosis among ODs has been reported 67.1% (6).

**Table 10:** Keywords related to respiratory disease.

**Keywords**	**1975–1990**	**1991–2006**	**2007–2021**
Occupational asthma	154	11	2
Asbestosis	48	6	0
Silicosis	20	0	7
Pneumoconiosis	12	0	10
Dust exposure	63	9	2
Chronic obstructive pulmonary	154	46	19
disease (COPD)			

The results of jobs analysis, which is influential in the type of occupational disease, show that 7 jobs were mentioned among the studies (Table 11). Among these, the most important job over the years has been health care workers. This job is mentioned in most occupational disease studies due to its high importance and numerous risks. On the other hand, the jobs of agricultural workers and hairdressers have been more frequent in the past years from 2007 to 2021 than other jobs, and this shows that the study of ODs is only for high-risk and well-known jobs such as construction and mining. Other jobs have been explored in recent years. In addition, the others section included occupations such as capacitor manufacturing workers, bakers, welders, firefighters, and kitchen employees, which were much less frequent than other occupations and could not be categorized separately. These results also indicate that the study of ODs should not be known only in the field of occupations, and future studies can also draw their results from ODs such as agricultural workers, hairdressers, capacitor manufacturing workers, bakers, welders, firefighters, and kitchen employees so that the diseases and risk factors of such occupations are better known.

**Table 11:** Keywords related to jobs.

**Jobs**	**1975–1990**	**1991–2006**	**2007–2021**
Health care workers	28	46	11
Construction workers	10	0	5
Miners	14	2	0
Agricultural workers	9	3	10
Chemical workers	4	2	4
Automobile industries	7	1	0
Hairdresser	0	2	6
Others	23	1	6

## Conclusion

The present study examined the main countries, institutions, and publications in the study of ODs and examined the interaction between them. This study also examined the keywords and research trends to find recent hot topics and predict future analysis trends. The conclusion of the present study is as follows:

1) From a study of the top 10 producing countries from 1975 to 2021, it can be seen that the United States and Germany are the two largest countries in producing studies on ODs. However, by analyzing Figures 6, 7, it can be concluded that Germany, with its current development trend in the coming years, will surpass the United States and gain the first rank.
2) The analysis of the cooperation of the top 10 countries showed that the level of cooperation between Spain is the highest, their cooperation reaches 1.40, and they have cooperated with 50 different countries. Also, Spain has the most cooperation (18 studies) with France, which has the highest number. This shows that the two countries have extensive international cooperation.
3) The results of the top 10 institutes show that the National Institute for Occupational Safety and Health (NIOSH) had the highest number of papers with 87 studies and Osnabrück University with 99 collaborations had the highest number of international collaborations. However, the survey showed that 3 out of the top 10 institutions were from Germany.
4) A study of keyword trends showed that researchers have paid more attention to skin diseases and mental health since 1991; this shows the importance of this issue in the study of ODs. Respiratory Diseases have also been ranked fourth among the keywords in three study periods and have always been of great importance.
5) In addition, job analysis shows that health care work has been the most important job during the past years, and then the jobs of agricultural workers and hairdressers in the past years from 2007 to 2021 have become more important than other jobs. Future studies can also report the results of ODs such as agricultural workers, hairdressers, capacitor manufacturing workers, bakers, welders, firefighters, and kitchen employees to better understand the diseases and risk factors of such occupations.
6) For the bibliometric analysis of the journals, the eigenfactor score can be used as an alternative bibliometrics that can help to choose beter journal. It is suggested to be used in future studies.

## Limitations

Our work clearly has some limitations. The most important one lies in the fact that since in scientometric studies in general and the software used, it is not possible to screen studies like what is done in systematic review studies (reading titiles, reading abstract, reading full text, etc), we must extract the most relevant studies by searching. Therefore, if only one disease is searched such as “pneumoconiosis,” a large number of studies will be found, many of which are not about occupational diseases. So, we tried to search the studies with a general keywords and only in the field of occupational diseases. By choosing this search strategy “Occupational disease^*^” OR “Occupational illness^*^” OR “Industrial disease^*^” OR “Industrial disease^*^” we tried to extract the most studies related to occupational diseases, regardless of the type of disease.

## Data availability statement

The original contributions presented in the study are included in the article/supplementary material, further inquiries can be directed to the corresponding author.

## Author contributions

MJ was the leader of study and edited the final manuscript. HS and HR gathered data and searched the articles and were a major contributor in writing the manuscript. MM and AZ analyzed the data and gathered the top tens and was a major contributor in writing the manuscript. All authors read and approved the final manuscript.

## Conflict of interest

The authors declare that the research was conducted in the absence of any commercial or financial relationships that could be construed as a potential conflict of interest.

## Publisher's note

All claims expressed in this article are solely those of the authors and do not necessarily represent those of their affiliated organizations, or those of the publisher, the editors and the reviewers. Any product that may be evaluated in this article, or claim that may be made by its manufacturer, is not guaranteed or endorsed by the publisher.
